# Supplementary material for: A Likelihood Approach for Real-Time Calibration of Stochastic Compartmental Epidemic Models
Source: PLoS Comput Biol. 2017 Jan 17;13(1):e1005257. doi: 10.1371/journal.pcbi.1005257 (PMC5240920; doi:10.1371/journal.pcbi.1005257)
Supplement: S1 File — (TAR.GZ) [file pcbi.1005257.s014.tar.gz › HSPH_Online-SI-Revision/IPoi09/n1-severe/n1-severe_BENCH2_table-8.pdf]

1	newly 3 weeks spec	19.	24.931	{0., 56.}	95.8258	0.728303	newly 3 weeks cumul	34.	94.8621	{0., 194.}	215.72	0.742296	newly 5 weeks spec	25.	12.7931	{0., 34.}	73.1034	1.14228	newly 5 weeks cumul	86.	125.586	{0., 240.}	95.83	0.659074	newly 62 weeks cumul	592.	147.483	{0., 300.}	75.0874	1.06991
2	newly 3 weeks spec	7.	60.8571	{14., 94.}	780.952	0.918551	newly 3 weeks cumul	19.	173.619	{36., 301.}	823.058	0.938841	newly 5 weeks spec	33.	56.9524	{11., 99.}	102.309	0.398494	newly 5 weeks cumul	67.	290.762	{86., 452.}	343.355	0.66231	newly 64 weeks cumul	626.	590.429	{186., 834.}	31.1882	0.262215
3	newly 3 weeks spec	17.	12.6111	{0., 45.}	95.098	1.28781	newly 3 weeks cumul	50.	54.1111	{2., 135.}	87.3333	0.618857	newly 5 weeks spec	23.	5.38889	{0., 37.}	91.0628	1.88582	newly 5 weeks cumul	88.	67.3889	{2., 156.}	73.4217	0.70884	newly 63 weeks cumul	618.	82.2778	{2., 257.}	86.6864	1.37171
4	newly 3 weeks spec	5.	15.1791	{0., 36.}	249.552	0.685765	newly 3 weeks cumul	13.	29.9403	{2., 72.}	177.842	0.510472	newly 5 weeks spec	23.	32.	{0., 75.}	107.268	0.712673	newly 5 weeks cumul	59.	84.8657	{2., 194.}	108.55	0.557652	newly 66 weeks cumul	656.	652.269	{2., 902.}	39.625	0.551397
5	newly 3 weeks spec	21.	13.7273	{0., 42.}	74.4589	0.899926	newly 3 weeks cumul	47.	31.5636	{0., 81.}	72.6886	0.840974	newly 5 weeks spec	31.	22.7273	{0., 59.}	81.7009	0.883003	newly 5 weeks cumul	110.	71.4909	{0., 222.}	73.9174	0.931845	newly 65 weeks cumul	613.	528.036	{0., 864.}	43.1351	0.850631
6	newly 3 weeks spec	13.	12.1364	{1., 26.}	69.5804	0.502199	newly 3 weeks cumul	22.	33.5227	{4., 78.}	106.921	0.440583	newly 5 weeks spec	15.	17.8409	{1., 38.}	90.7576	0.559953	newly 5 weeks cumul	45.	65.8182	{8., 149.}	104.141	0.479768	newly 62 weeks cumul	650.	551.955	{229., 734.}	22.2028	0.296221
7	newly 3 weeks spec	23.	33.7963	{5., 67.}	88.1643	0.423041	newly 3 weeks cumul	69.	90.1481	{11., 182.}	82.0719	0.423751	newly 5 weeks spec	34.	39.8148	{5., 68.}	63.6166	0.412039	newly 5 weeks cumul	132.	167.185	{23., 328.}	72.9798	0.398832	newly 62 weeks cumul	490.	627.296	{340., 799.}	44.1497	0.276039
8	newly 3 weeks spec	11.	23.6557	{0., 62.}	171.982	0.702878	newly 3 weeks cumul	28.	47.5738	{1., 121.}	132.904	0.565676	newly 5 weeks spec	39.	46.4262	{0., 112.}	79.4872	0.660617	newly 5 weeks cumul	95.	130.18	{1., 346.}	97.4633	0.593375	newly 66 weeks cumul	644.	693.787	{1., 909.}	42.2284	0.5743
9	newly 3 weeks spec	13.	39.5965	{7., 69.}	224.831	0.541641	newly 3 weeks cumul	27.	95.4035	{15., 183.}	272.71	0.604018	newly 5 weeks spec	24.	53.8772	{6., 82.}	150.95	0.492013	newly 5 weeks cumul	73.	194.947	{32., 330.}	189.354	0.543202	newly 62 weeks cumul	503.	718.737	{642., 841.}	53.7651	0.32129
10	newly 3 weeks spec	19.	38.8485	{6., 71.}	133.333	0.46073	newly 3 weeks cumul	44.	90.0909	{14., 185.}	134.986	0.430009	newly 5 weeks spec	47.	55.1212	{10., 84.}	53.7718	0.345822	newly 5 weeks cumul	144.	192.03	{30., 323.}	70.4545	0.360437	newly 62 weeks cumul	637.	756.591	{657., 861.}	28.8307	0.219518
11	newly 3 weeks spec	13.	36.625	{9., 65.}	197.39	0.495706	newly 3 weeks cumul	42.	98.1429	{27., 176.}	150.595	0.417906	newly 5 weeks spec	27.	41.5179	{19., 67.}	71.8915	0.281685	newly 5 weeks cumul	89.	179.482	{63., 301.}	117.436	0.375441	newly 59 weeks cumul	567.	601.643	{337., 761.}	24.7858	0.179751
12	newly 3 weeks spec	17.	13.2113	{0., 31.}	100.	1.03358	newly 3 weeks cumul	30.	24.1549	{1., 59.}	93.1925	0.779446	newly 5 weeks spec	56.	28.8028	{0., 96.}	84.6831	1.27279	newly 5 weeks cumul	124.	73.6901	{1., 191.}	87.6647	1.01935	newly 67 weeks cumul	637.	489.803	{1., 915.}	56.6586	1.06185
13	newly 3 weeks spec	11.	30.9412	{4., 67.}	211.586	0.537226	newly 3 weeks cumul	31.	75.549	{10., 151.}	174.7	0.492075	newly 5 weeks spec	30.	38.9608	{4., 69.}	68.6928	0.38833	newly 5 weeks cumul	95.	149.196	{22., 286.}	95.356	0.41927	newly 62 weeks cumul	650.	626.314	{424., 795.}	20.6335	0.235401
14	newly 3 weeks spec	13.	5.45205	{0., 15.}	90.5163	1.22378	newly 3 weeks cumul	26.	10.8219	{0., 34.}	86.4067	1.04581	newly 5 weeks spec	24.	11.6712	{0., 32.}	101.142	1.47226	newly 5 weeks cumul	70.	30.2192	{0., 81.}	92.0157	1.2433	newly 67 weeks cumul	660.	344.384	{0., 876.}	62.7978	1.45131
15	newly 3 weeks spec	34.	60.84	{23., 87.}	90.4706	0.284455	newly 3 weeks cumul	78.	178.58	{71., 260.}	134.231	0.349333	newly 5 weeks spec	40.	51.28	{20., 80.}	52.8	0.232336	newly 5 weeks cumul	154.	287.78	{116., 377.}	94.6364	0.283925	newly 61 weeks cumul	487.	535.9	{274., 771.}	32.8583	0.154518
16	newly 3 weeks spec	11.	37.5	{0., 111.}	305.584	0.880305	newly 3 weeks cumul	21.	72.6143	{0., 203.}	309.048	0.845152	newly 5 weeks spec	31.	54.9	{0., 124.}	148.341	0.864184	newly 5 weeks cumul	74.	176.486	{0., 463.}	205.985	0.877498	newly 67 weeks cumul	620.	649.514	{0., 919.}	46.9954	0.716264
17	newly 3 weeks spec	34.	32.3529	{0., 99.}	97.4048	0.918035	newly 3 weeks cumul	57.	58.5147	{1., 187.}	97.807	0.748223	newly 5 weeks spec	41.	60.1471	{0., 161.}	119.727	0.805008	newly 5 weeks cumul	126.	166.515	{1., 474.}	109.419	0.760004	newly 67 weeks cumul	668.	627.029	{1., 919.}	42.5898	0.674517
18	newly 3 weeks spec	10.	6.31579	{0., 16.}	91.8421	0.995756	newly 3 weeks cumul	22.	12.9079	{0., 35.}	80.2033	0.8392	newly 5 weeks spec	22.	16.1316	{0., 69.}	98.2057	1.14008	newly 5 weeks cumul	59.	40.1711	{0., 134.}	89.2284	0.982073	newly 67 weeks cumul	630.	481.526	{0., 894.}	55.3049	1.10751
19	newly 3 weeks spec	31.	36.4146	{11., 55.}	52.085	0.277066	newly 3 weeks cumul	69.	112.22	{44., 173.}	84.4115	0.352269	newly 5 weeks spec	48.	31.2683	{9., 51.}	40.0407	0.383045	newly 5 weeks cumul	148.	180.098	{70., 263.}	47.3632	0.27439	newly 60 weeks cumul	633.	356.78	{129., 565.}	45.5477	0.421387
20	newly 3 weeks spec	33.	33.4667	{6., 61.}	50.6061	0.327018	newly 3 weeks cumul	87.	82.3667	{18., 148.}	49.5785	0.317582	newly 5 weeks spec	54.	42.9667	{8., 69.}	35.1235	0.271339	newly 5 weeks cumul	182.	163.367	{37., 274.}	42.7839	0.297256	newly 60 weeks cumul	622.	695.15	{556., 810.}	18.5557	0.130648
21	newly 3 weeks spec	27.	100.574	{0., 171.}	306.132	0.876492	newly 3 weeks cumul	47.	278.902	{0., 499.}	526.195	1.0728	newly 5 weeks spec	26.	71.9836	{0., 148.}	217.213	0.783109	newly 5 weeks cumul	97.	441.262	{0., 695.}	384.823	1.03221	newly 67 weeks cumul	609.	621.393	{0., 896.}	44.4319	0.682032
22	newly 3 weeks spec	20.	24.3333	{2., 55.}	85.	0.516555	newly 3 weeks cumul	51.	63.2381	{5., 139.}	84.5005	0.443666	newly 5 weeks spec	65.	24.7857	{2., 47.}	62.0147	0.708486	newly 5 weeks cumul	155.	113.905	{12., 229.}	54.8694	0.471439	newly 61 weeks cumul	627.	553.024	{294., 778.}	28.2562	0.293893
23	newly 3 weeks spec	22.	48.9714	{4., 92.}	151.688	0.560449	newly 3 weeks cumul	61.	150.857	{15., 258.}	170.632	0.571281	newly 5 weeks spec	31.	36.4286	{0., 73.}	83.5023	0.615178	newly 5 weeks cumul	113.	230.029	{16., 377.}	127.889	0.527757	newly 63 weeks cumul	543.	320.686	{16., 570.}	46.4036	0.540604
24	newly 3 weeks spec	19.	60.8571	{26., 93.}	226.128	0.507224	newly 3 weeks cumul	60.	199.625	{99., 294.}	236.994	0.54156	newly 5 weeks spec	37.	44.25	{14., 78.}	57.0463	0.283954	newly 5 weeks cumul	115.	296.089	{146., 421.}	163.059	0.442413	newly 60 weeks cumul	485.	453.768	{174., 722.}	36.3513	0.246939
25	newly 3 weeks spec	31.	16.3704	{0., 43.}	59.8566	0.748101	newly 3 weeks cumul	67.	43.8148	{3., 98.}	62.9077	0.634037	newly 5 weeks spec	70.	15.2222	{0., 35.}	78.254	1.09405	newly 5 weeks cumul	176.	74.5926	{3., 176.}	59.2593	0.760014	newly 58 weeks cumul	653.	388.407	{3., 590.}	40.5195	0.620646
26	newly 3 weeks spec	28.	15.0179	{0., 41.}	70.0893	0.969866	newly 3 weeks cumul	57.	35.625	{0., 95.}	72.2744	0.952504	newly 5 weeks spec	43.	24.1786	{0., 69.}	70.515	0.97572	newly 5 weeks cumul	130.	79.8393	{0., 240.}	71.2775	0.994946	newly 65 weeks cumul	587.	510.929	{0., 816.}	47.4203	0.959844
27	newly 3 weeks spec	19.	71.029	{20., 114.}	280.397	0.558319	newly 3 weeks cumul	36.	160.522	{41., 298.}	352.013	0.610546	newly 5 weeks spec	37.	96.1739	{62., 127.}	163.063	0.43741	newly 5 weeks cumul	94.	341.435	{140., 499.}	267.268	0.567005	newly 64 weeks cumul	546.	828.986	{684., 920.}	54.738	0.236539
28	newly 3 weeks spec	3.	6.26087	{0., 22.}	205.314	0.868306	newly 3 weeks cumul	9.	12.3478	{0., 39.}	132.528	0.816511	newly 5 weeks spec	27.	12.2899	{0., 50.}	91.9485	1.39238	newly 5 weeks cumul	65.	33.7101	{0., 116.}	87.6923	1.22496	newly 67 weeks cumul	649.	349.029	{0., 847.}	61.401	1.49259
29	newly 3 weeks spec	26.	43.193	{11., 67.}	88.664	0.309226	newly 3 weeks cumul	77.	120.14	{37., 193.}	82.4561	0.301322	newly 5 weeks spec	43.	44.5088	{16., 65.}	34.1901	0.188888	newly 5 weeks cumul	154.	208.211	{61., 309.}	59.6719	0.244925	newly 60 weeks cumul	551.	567.088	{354., 756.}	22.8006	0.121699
30	newly 3 weeks spec	38.	26.8	{0., 61.}	62.1053	0.722107	newly 3 weeks cumul	100.	93.8333	{4., 179.}	63.5667	0.526851	newly 5 weeks spec	52.	18.1333	{0., 40.}	68.7179	1.13903	newly 5 weeks cumul	190.	134.033	{4., 279.}	53.7368	0.57465	newly 61 weeks cumul	601.	208.467	{4., 443.}	65.3134	0.82504
31	newly 3 weeks spec	14.	46.0769	{15., 73.}	241.209	0.549183	newly 3 weeks cumul	56.	131.385	{40., 215.}	149.725	0.424046	newly 5 weeks spec	13.	48.8462	{12., 73.}	288.166	0.60082	newly 5 weeks cumul	98.	230.519	{113., 344.}	146.801	0.434468	newly 62 weeks cumul	505.	555.923	{268., 766.}	33.4349	0.207904
32	newly 3 weeks spec	13.	19.434	{1., 38.}	105.225	0.541385	newly 3 weeks cumul	24.	42.5094	{4., 91.}	123.349	0.459634	newly 5 weeks spec	8.	31.0566	{0., 60.}	324.057	0.74046	newly 5 weeks cumul	57.	97.6792	{5., 197.}	119.762	0.497708	newly 64 weeks cumul	509.	676.585	{25., 868.}	55.2767	0.414535
33	newly 3 weeks spec	19.	23.7538	{0., 78.}	119.595	0.831209	newly 3 weeks cumul	30.	43.8	{1., 130.}	133.59	0.697996	newly 5 weeks spec	36.	43.6308	{0., 122.}	99.4872	0.841336	newly 5 weeks cumul	97.	123.323	{1., 360.}	110.658	0.771513	newly 67 weeks cumul	689.	629.523	{1., 925.}	41.63	0.731151
34	newly 3 weeks spec	23.	45.7955	{19., 77.}	107.016	0.287972	newly 3 weeks cumul	76.	157.727	{89., 236.}	111.842	0.304632	newly 5 weeks spec	29.	36.4318	{5., 63.}	68.2602	0.301931	newly 5 weeks cumul	132.	234.	{119., 355.}	84.4008	0.250203	newly 59 weeks cumul	459.	361.977	{138., 598.}	35.1802	0.216854
35	newly 3 weeks spec	15.	43.127	{0., 108.}	233.651	0.71457	newly 3 weeks cumul	32.	79.873	{1., 187.}	197.024	0.633671	newly 5 weeks spec	45.	83.7143	{0., 168.}	125.608	0.672183	newly 5 weeks cumul	99.	228.571	{1., 513.}	170.13	0.687123	newly 67 weeks cumul	642.	715.841	{1., 910.}	42.6	0.571351
36	newly 3 weeks spec	67.	29.85	{0., 80.}	96.9403	1.72762	newly 3 weeks cumul	106.	50.83	{0., 159.}	98.8208	1.6234	newly 5 weeks spec	61.	41.04	{0., 146.}	103.672	1.66562	newly 5 weeks cumul	222.	129.98	{0., 477.}	98.8829	1.70648	newly 68 weeks cumul	649.	405.63	{0., 917.}	66.0971	1.69372
37	newly 3 weeks spec	27.	47.3061	{23., 70.}	85.941	0.272714	newly 3 weeks cumul	77.	136.204	{61., 205.}	88.9213	0.288062	newly 5 weeks spec	49.	41.102	{19., 69.}	31.6951	0.176018	newly 5 weeks cumul	165.	221.816	{112., 318.}	48.7817	0.192038	newly 58 weeks cumul	598.	465.347	{257., 638.}	25.657	0.145904
38	newly 3 weeks spec	10.	50.9242	{9., 123.}	425.909	0.692505	newly 3 weeks cumul	38.	98.8182	{12., 238.}	192.743	0.553236	newly 5 weeks spec	51.	88.0455	{17., 134.}	100.624	0.459606	newly 5 weeks cumul	123.	262.136	{40., 506.}	140.983	0.532995	newly 66 weeks cumul	597.	788.227	{520., 925.}	45.985	0.372259
39	newly 3 weeks spec	24.	27.8696	{1., 62.}	75.7246	0.481503	newly 3 weeks cumul	41.	72.6304	{7., 145.}	119.247	0.458127	newly 5 weeks spec	32.	35.1087	{0., 66.}	67.4592	0.525837	newly 5 weeks cumul	105.	138.761	{17., 272.}	78.1573	0.425348	newly 62 weeks cumul	520.	547.739	{58., 818.}	43.3863	0.401458
40	newly 3 weeks spec	29.	8.59	{0., 22.}	89.8966	1.61922	newly 3 weeks cumul	61.	14.01	{0., 40.}	87.1639	1.5555	newly 5 weeks spec	48.	23.2	{0., 77.}	96.375	1.72849	newly 5 weeks cumul	145.	52.98	{0., 129.}	90.2759	1.70074	newly 68 weeks cumul	580.	345.19	{0., 917.}	75.2397	1.81181
41	newly 3 weeks spec	22.	26.8125	{1., 58.}	83.0966	0.49863	newly 3 weeks cumul	38.	55.2188	{5., 127.}	102.549	0.485376	newly 5 weeks spec	81.	46.75	{1., 94.}	51.8904	0.609972	newly 5 weeks cumul	156.	138.172	{6., 286.}	58.0829	0.50965	newly 65 weeks cumul	649.	739.641	{334., 918.}	36.212	0.399769
42	newly 3 weeks spec	18.	82.9107	{16., 134.}	374.702	0.723268	newly 3 weeks cumul	47.	204.946	{47., 354.}	349.962	0.711397	newly 5 weeks spec	26.	86.1607	{9., 123.}	250.069	0.641118	newly 5 weeks cumul	102.	380.839	{87., 571.}	286.642	0.69374	newly 65 weeks cumul	610.	707.911	{194., 901.}	38.709	0.350416
43	newly 3 weeks spec	35.	24.6538	{5., 45.}	46.4835	0.39059	newly 3 weeks cumul	83.	65.9231	{13., 133.}	48.3318	0.349397	newly 5 weeks spec	42.	28.5769	{5., 49.}	38.6447	0.32227	newly 5 weeks cumul	155.	122.442	{30., 215.}	42.2705	0.313254	newly 58 weeks cumul	514.	597.731	{474., 720.}	23.9823	0.138143
44	newly 3 weeks spec	15.	40.9839	{3., 92.}	211.075	0.587776	newly 3 weeks cumul	34.	84.3871	{9., 191.}	188.994	0.55793	newly 5 weeks spec	30.	63.871	{4., 116.}	146.344	0.547872	newly 5 weeks cumul	77.	203.371	{25., 420.}	196.628	0.592544	newly 66 weeks cumul	567.	762.355	{528., 907.}	52.1363	0.408748
45	newly 3 weeks spec	32.	57.4182	{15., 96.}	101.705	0.368136	newly 3 weeks cumul	68.	131.909	{35., 250.}	117.834	0.368335	newly 5 weeks spec	56.	75.5455	{36., 103.}	51.6558	0.217336	newly 5 weeks cumul	159.	275.418	{107., 419.}	92.4528	0.319615	newly 63 weeks cumul	632.	761.255	{611., 896.}	24.813	0.0974934
46	newly 3 weeks spec	21.	31.6724	{1., 63.}	89.7373	0.479311	newly 3 weeks cumul	37.	85.8621	{7., 173.}	160.857	0.536163	newly 5 weeks spec	19.	37.5517	{5., 63.}	123.956	0.490428	newly 5 weeks cumul	68.	159.741	{16., 277.}	160.877	0.564039	newly 62 weeks cumul	566.	585.069	{209., 779.}	29.3286	0.352671
47	newly 3 weeks spec	13.	29.85	{0., 80.}	256.538	1.33343	newly 3 weeks cumul	21.	50.83	{0., 159.}	262.143	1.23611	newly 5 weeks spec	26.	41.04	{0., 146.}	182.385	1.50027	newly 5 weeks cumul	64.	129.98	{0., 477.}	225.563	1.45576	newly 68 weeks cumul	615.	405.63	{0., 917.}	68.7756	1.6896
48	newly 3 weeks spec	37.	69.7246	{23., 98.}	105.366	0.342105	newly 3 weeks cumul	81.	172.71	{45., 264.}	131.079	0.381317	newly 5 weeks spec	34.	77.6087	{38., 104.}	137.127	0.383295	newly 5 weeks cumul	153.	322.841	{119., 448.}	124.003	0.364588	newly 62 weeks cumul	483.	759.072	{651., 860.}	57.1579	0.193477
49	newly 3 weeks spec	13.	37.625	{0., 81.}	231.25	0.720148	newly 3 weeks cumul	42.	83.0781	{0., 234.}	153.013	0.731262	newly 5 weeks spec	46.	57.1094	{0., 117.}	78.2948	0.653892	newly 5 weeks cumul	113.	186.031	{0., 399.}	114.298	0.756067	newly 66 weeks cumul	632.	652.016	{0., 913.}	44.3063	0.727784
50	newly 3 weeks spec	9.	26.7308	{0., 61.}	235.043	0.669847	newly 3 weeks cumul	25.	62.9808	{2., 127.}	189.462	0.637186	newly 5 weeks spec	19.	39.6731	{0., 80.}	155.769	0.646318	newly 5 weeks cumul	58.	134.558	{2., 282.}	172.447	0.67335	newly 64 weeks cumul	488.	585.731	{2., 823.}	55.1151	0.584854
